# Youth are united online to fight against involution: a study of group cohesion on Weibo

**DOI:** 10.3389/fpsyg.2023.1014331

**Published:** 2023-05-18

**Authors:** Yang Zhang, Tong Ji

**Affiliations:** ^1^School of New Media and Communication, Tianjin University, Tianjin, China; ^2^Peking University Library, Beijing, China

**Keywords:** generation gap, group cohesion, involution, public opinion online, STM, BERT, hashtag, social identity

## Abstract

**Background:**

In China, involution, which means pressure to out-compete other group members, has attracted public attention on Weibo. The new online connotation of involution empowered group cohesion among youth. Dissimilar to other crises, this crise also closely relates to group cohesion concept. However, few previous group cohesion-related studies focus on this critical concept. This study explains why and how youth created group cohesion online when facing involution. First, by examining the relationship between involution and group cohesion. Second, by examining whether youth are united in the online discussion of involution by investigating the generational gap. Following this, this study analyzes the different opinions to identify why this group cohesion occurs, how youth think about involution, and why they regard “older adults” as others. Lastly, this study analyzes how youth use hashtags to attract more youth to voice their opinions, consequently leading to greater group cohesion.

**Methods:**

By combining frontier computational methods with causation and axial coding, this study proposes a new way to in-depth analyze group cohesion on social media.

**Results:**

The results indicate that involution triggers poor online group cohesion, and online involution-related hot issues trigger identity-based group cohesion. Additionally, youth are significantly more negative than older adults, and their expressions are full of identity-based construction. By stressing the social roots and blaming the “other” (older adult group), youth united together online. These findings indicated that a generation gap does indeed exist and that youth unite on social media by posting related hashtags via “revealing social identity” and “positioning and becoming” strategies.

**Conclusion:**

The findings stress that involution is related to poor group cohesion and that social media offers a new way to face the involution crisis. Youth will use hashtags to unite and blame imagined enemies, such as older adults and the upper class. These findings might assist in understanding interventions that lead to more group cohesion.

## 1. Introduction

At the end of 2020, a picture attracted public attention: a Tsinghua University student rode a bike while holding a laptop and staring at it. Other students explained that “Wasting time on things like riding a bike becomes unacceptable due to the intense pressure of graduating top of the class.” Chinese citizens started to discuss a new buzzword on Weibo: “neijuan” (involution).

Involution was introduced as a social concept by American sociologist Clifford Geertz to describe a process of self-circulation and stagnation of development where the same behavior is repeated over a long time and maintained at a certain level without any changes or promotions (Liu and Qiu, 2004). Later, this concept was adopted by more and more scholars in different areas to describe social phenomena (Kang and Jin, 2020). Duara (1988) used the term “involution” to describe being stuck in the old system, which only caused quantitative changes.

The word has been discussed widely by the public since the end of 2020, and its new connotation, as vigorous as a kind of self-generated concept by social media, has been used as attribution to endless fierce in-group competition, which may bring personal and social stagnancy (Wang and Ge, 2020). This endless fierce in-group competition appeared in several countries as a social phenomenon (Lindquist and Xiang, 2019). For example, research conducted in the United States indicated that four in ten millennials aged 25–37 have a bachelor’s degree or above but earn less than the previous generation of the same education level. Industrialized East Asian countries such as Japan and South Korea, where an aging population compounds economic stagnation, have seen some of the worst involutions for some time. In 2020, by discussing “involution,” Chinese individuals discussed whether endless fierce competition for youth groups could make their lives better since they were facing the fact that opportunities for youth were decreasing.

In fierce competition games, youth have bad relationships with peers. According to a survey released by the Center for Mental Health of the Chinese Center for Disease Control and Prevention, 35% of mental health disorders are related to a “comparative crisis” in which individuals “find themselves inferior to others.” Youth start to voice their opinions on social media, for example, using hashtags, to search for resonance and fight against involution.

Dissimilar to other crises, involution means irrational competition ingroup, which also relates to group cohesion. To the best of our knowledge, few group cohesion-related studies focus on this critical concept. This study attempted to explain how youth recreated group cohesion online when facing involution, the offline fierce peer competition game. This study first examined the relationship between involution and group cohesion. Following this, this study examined whether youth were united in the online involution discussion by detecting possible generational gaps. Additionally, different opinions were analyzed to determine why this group cohesion exists, how youth think about involution, and why they regard “older adults” as others. Lastly, this study analyzed how youth used hashtags to attract more group members to voice their opinions.

## 2. Literature review

### 2.1. Involution and group cohesion online

The term “involution” describes a culture that cannot (or does not) adapt and or expand its economy but continues to develop in the direction of internal complexity and inefficiency (Hui, 2009). Geertz (1963) used this term to describe the decline of agriculture in Java. Further, “involution” is used to describe sociopolitical problems in opposition to “evolution” (Santosa et al., 2013).

Some scholars believe that involution should be seen as an administrative setback, dwarfing autonomous regions with the intention of power-sharing. The parent region is considered uncaring toward grassroots welfare, is discriminatory, and creates development gaps (Smail, 1965). Accelerated development of parent regions must be dwarfed, and power-sharing requires grassroots approval by consolidating identity (Haboddin, 2012). Duara (1988) used the term “involution” to describe being stuck in the old system, which only caused quantitative changes. In his book, Duara believed that involution is not limited to agriculture and the economy and can further foreground other areas such as sociology and psychology. He stated that involution refers to growth without improving efficiency and actual development; it depends on the regeneration and survival of the old ways.

These implications suggested that meaningless in-group competition should be critical in the “involution” concept. In 2020, “involution” became one of the most popular words online, describing the meaningless competition in all aspects of individuals’ daily lives, including career development, students’ performance improvement, etc. Its new connotation, as vigorous as a kind of self-generated concept by social media, has been used as attribution to any peer competition pressures causing either personal or social stagnancy.

Kang and Jin (2020) believed that the concept can serve as an essential indicator for behavioral studies at individual and group levels. They stated that involution has a significant impact on personal growth and can be understood by higher and lower needs from Maslow’s hierarchy of needs perspective. Involution is helpful to indicate whether our lives are in such an inefficient in-group competition cycle.

It is inevitable to consider group cohesion when discussing involution. Festinger (1950) defined group cohesion as the result of all the forces acting on the members to remain in the group (p. 274). Sargent and Sue-Chan (2001) measured group cohesion from four dimensions: “I liked my group,” “I got along with members of my group,” “I am friends with the members of my group,” and “I feel a sense of belongingness to my group.” As the concept refers to the extent of connectedness and solidarity among groups in society (Kawachi and Berkman, 2000), group cohesion is related to in-group competition (e.g., Forsyth and Kolenda, 1966; Lyles et al., 2018; Newson et al., 2022), and thus connected to involution. According to Lyles et al. (2018), friendly competition and cooperation are consistent strong predictors of cohesion. That is to say, the prevalence of involution, which refers to fierce and endless in-group competition, might lead to poor group cohesion.

Traditionally, the psychological literature on intergroup conflict was strongly influenced by social identity, which emphasizes the relationship between in-group favoritism and out-group derogation (Tajfel et al., 1979). Individuals who share the same social categories such as sex, country, occupation, and age may share their attitudes online. Group cohesion has also been linked to various dimensions of well-being (Cramm et al., 2013; Yu et al., 2019). Additionally, according to self-categorization theory (Turner and Reynolds, 1987), increased group cohesion can directly result from a specific collective identity being activated by contextual changes such as particular hot issues driven by intergroup conflict. Intergroup conflict highlights self-categorizations in terms of ‘us versus them,’ and hot issues underpinned by such conflict can provide the group with more explicit goals, a shared vision, and clear guidelines for behaviors in alignment with group norms (Bliuc et al., 2015). The specific conditions created by intergroup conflict may help opposing groups identify their respective group’s enemy more clearly and thus provide a concrete basis for identity formation, increased identification in-group (Schmid and Muldoon, 2015; Stott et al., 2017).

In ICT times, social media makes it easier for individuals to strengthen their group cohesion (Bliuc et al., 2020). Compared to small groups, larger groups, related to the same social categories in different places, unite to share the same views and show their support to each other. For example, women stay together and voice for each other online when facing harassment offline (Portwood-Stacer and Berridge, 2014). Group cohesion is formulated, and their voices might differ from men due to different sex cultures and experiences. Communication researchers proved that having a large number of user-generated social data available is an excellent opportunity to investigate the communicative behaviors emerging in the context of debates and to shed light on how communities of users with different roles in society and different sentiments interact (Lai et al., 2015). Thus, it is plausible that if group cohesion appears online, a gap can be found between different socially categorized groups. Although the opinion gap between different social groups reveals social-identity-related group cohesion, only a few studies (e.g., Zhu et al., 2022) examined it from a group cohesion perspective. Thus, the polarized opinions between larger social-identity-based groups may reflect a union of group members online, and the way group members attract voices from other group members strengthens group cohesion online and offline.

However, the discussion of involution on Weibo is unique because it discusses in-group competition and the poor group cohesion situation, and individuals are debating online with more and more polarized opinions. For example, some felt desperate about fierce in-group competition, while some were more positive and encouraged to join the competition. If the polarized opinions about involution online are based on social categories, the public opinion and the way they unite together are both valuable to understanding the strengthened group cohesion.

### 2.2. Generation gap in involution-related discussion online

This section discusses why this study predicts that group cohesion would appear among generational groups when discussing involution.

Several studies have illuminated the nature and extent of continuity or differences between age groups, which refers to the “generation gap” (Bengtson, 1970). According to Kupperschmidt (2000), a generation is an identifiable group that shares birth, years, age, and significant life events at critical developmental stages (p. 66). A generation of employees consists of individuals born approximately within the same period of one to two decades (Kupperschmidt, 2000).

Scholars have specifically concentrated on the generation gap in perception and expectation toward work. The American Water Works Association reported that multiple generations are arguing together (Wood, 2005). This has resulted in a level of generational conflict that is so pervasive, it influences how employees are treated (competition rules in workplace), how career goals are met. Additionally, studies indicated that conflicts between age groups toward work can be a social problem in most Western industrialized countries (see Neugarten, 1970).

Previous studies have discussed the reason why the generation gap in perception and expectation toward competition in workplace exists. Scholars believe that when individuals from the same generation share similar historical, economic, and social experiences, they have similar work values, attitudes, and behaviors (Zemke et al., 2000; Smola and Sutton, 2002). Gursoy et al. (2008, p. 450) stressed that members of generations who come of age during lean times or war years tend to think and act differently than those born in peace and abundance. Therefore, the significant life experiences of individuals belonging to each generational group tend to shape their unique characteristics, aspirations, and expectations (Cennamo and Gardner, 2008). Scholars also consider social change, life pathways, and individual development as modes of behavioral continuity and change, consequently believing that different age groups may have different opinions about competition in the workplace (Yang et al., 2008; Eber et al., 2021). Regarding students, involution is one of the most popular words related to competition in recent studies. Parents and students may also share different opinions due to their various situations when facing involution (Ghiocanu, 2016).

Compared to older adults, only young people get involved in the fierce in-group competition game, involution, in China. Starting from school, they must keep taking exams to compete with their peers. After starting work, they need job promotions to make more money. Young Chinese individuals are also eager to buy a house, however, the high and increasing prices forces youth to work harder to obtain more money. Consequently, it is impossible to have an easy life and compete as before. As mentioned above, many societies face social stagnancy, which makes youth stuck in endless competition without actual improvement to themselves or society. Therefore, compared to older adults, the out-group members, who have settled down or have a high social status in China, young individuals suffer from involution. This also provided a basis for the different attitudes of different age groups toward involution.

Not restricted to workplace or school offline, one’s opinion can appeal to resonance from other same generational group members online. Studies have found that attitude is an excellent way to measure the generation gap (Duncan, 1978), particularly toward peer competition (Eber et al., 2021). Attitudes are a tendency to evaluate a target object with some degree of favor or disfavor. They can be operationalized by emotional reactions, behaviors toward, and cognitive evaluations of the object (Netzer et al., 2018). Our paper finds attitudes toward involution as opinions of involution and sentiment (favor or disfavor). Whether the attitudes are varied among different generational groups online can detect the existence of group cohesion; the analysis of involution-related opinions reveals what different generations think about in-group competition and group cohesion; and how they attract same-social-category group members to voice online explains how group cohesion is strengthened online. However, little research has been conducted on group cohesion using this perspective.

Hashtag usage can also reveal how generational group cohesion is strengthened online. Cappellini et al. (2019) stated that we are presently witnessing groups formulating not only in communities but among the wider public on social media, where several hashtags are increasingly used to divide, influence, and agitate (Gillespie et al., 2014; Pasquale, 2015; Striphas, 2015). Research into the use of hashtags on Twitter and other social media platforms developed rapidly following the Arab Spring and Occupy protests that foregrounded the role of hashtags in informing the public, coordinating campaigns, and expressing support (e.g., Papacharissi and de Fatima Oliveira, 2012; Bruns et al., 2013). Studies explored the phenomenon of hashtags and their significance in disseminating news, communicating with dispersed publics during crisis events, and in publicity efforts by celebrities, politicians, or charities (Portwood-Stacer and Berridge, 2014). Additionally, studies examined how hashtags raise awareness and shape public attitudes on various social issues, events, and ideas (e.g., Rodgers and Scobie, 2015; Dragiewicz and Burgess, 2016; Stout, 2016). Studies revealed that hashtags are not only markers indicating a gathering of like-minded individuals (e.g., Sunstein, 2018) but are amplifiers, flagging crucial issues for a wider public and making them discussable (Stout, 2016). Thus, it is a powerful way to enlarge social groups online. Studies further indicated that hashtags are constituted in multifarious exchanges, thus creating complex dynamics of collaboration, solidarity, and confrontation (Cappellini et al., 2019). Researchers suggest that viewing hashtags as borderscapes enables a more nuanced understanding of opinion polarization (Cappellini et al., 2019), the processes inhibiting encounters with social-cultural others (Bump, 2016; Sunstein, 2018).

The present study aimed to examine online group cohesion via hashtags. The hashtags used online by youth can be used to analyze how youth appeal to resonance to their social identity-related group and strengthen their group cohesion. This study explored the usage of the hashtag in denoting the contours of diverse positions on the contested social issue involution.

**Figure 1:**
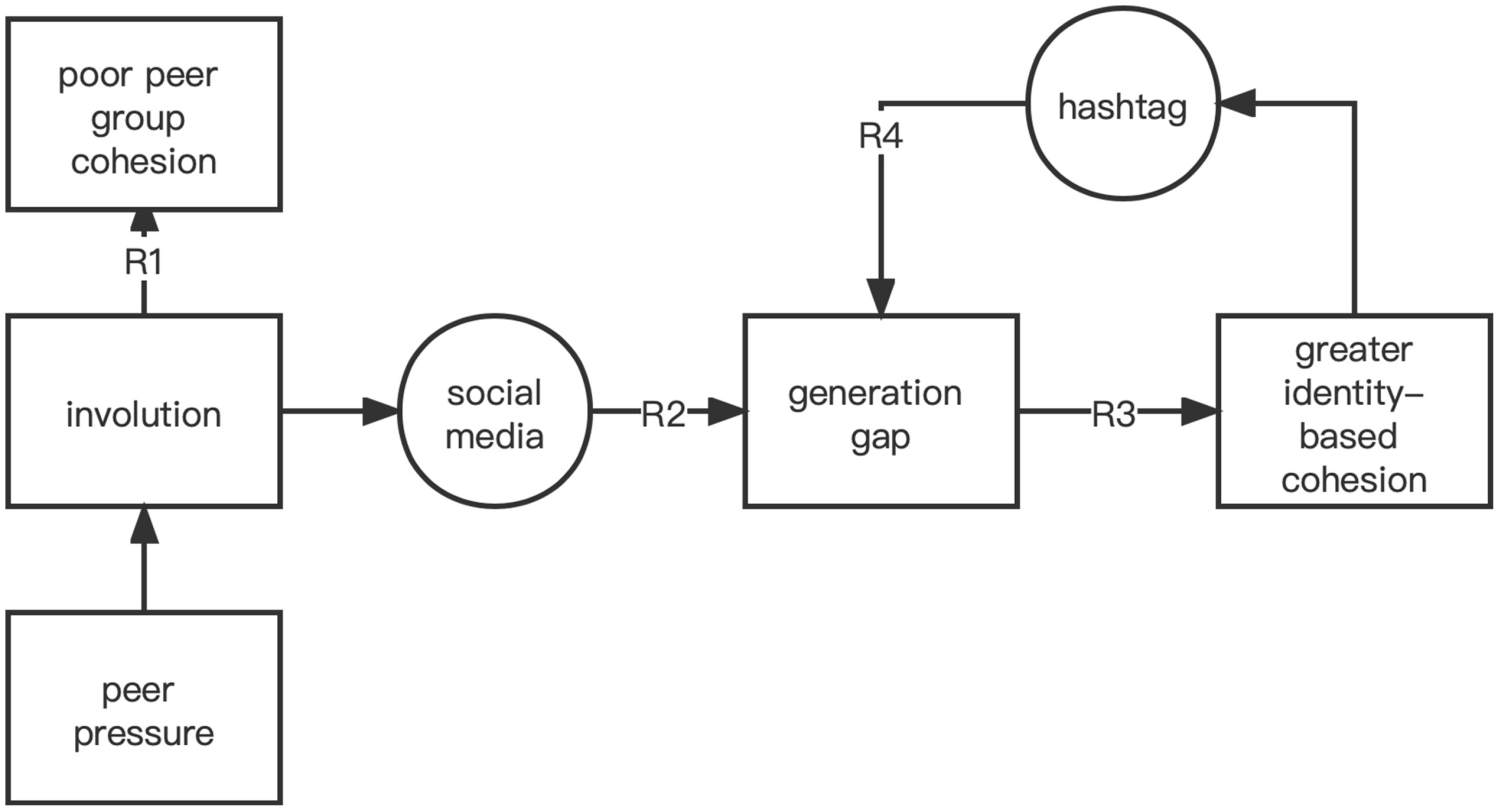
The research flow.

This study attempted to understand how youth use social media to unite and fight against involution (shown in Figure 1). First, the relationship between involution and group cohesion was examined (RQ1). Second, whether youth were united in the discussion of involution online was examined by detecting possible generational gaps (RQ2). Third, different opinions were analyzed to identify why group cohesion increase among youth when discussing involution (RQ3). Finally, how youth used hashtags to attract more group members to voice and achieve greater group cohesion was analyzed (RQ4).

This study’s first question focused on the relationship between involution and group cohesion:

RQ1: What did youth say about their group cohesion in real life when discussing involution? Does the involution cause poor group cohesion?

Starting from the literature on the generation gap in peer competition offline, this study detected different attitudes toward involution among different generational groups online. Thus, the second research question was as follows:

RQ2: Do different generational groups have different attitudes toward involution on Weibo?

The unity of identity-based groups online can be seen from several perspectives, such as distinguishing “them” from “us,” and having different opinions compared to other groups. If differences were present, the differences of opinions toward involution were another focus of the generation gap. From the differences, the relationship between involution and group cohesion and whether the out-group members force the youth to unite online could be understood. Thus, the third research question was as follows:

RQ3: What are the different opinions between youth and older adult groups when discussing involution on Weibo?

Finally, hashtags used online by youth were analyzed to understand how youth appeal to resonance to their social identity-related group and strengthen their group cohesion using ICT. Thus, the fourth research question was as follows:

RQ4: What are the hashtags youth use when discussing involution on Weibo? How do they frame the hashtag?

## 3. Method

By analyzing social data related to involution collected from October 13, 2020, to September 13, 2021, consisting of 21 thousand Weibo posts, this study detected the sentimental divide using Bidirectional Encoder Representations from Transformers (BERT) and Structural Topic Model (STM) methods.

With neural networks’ capacity for learning representation from data, BERT performs excellently in sentence-level sentiment classification. Thus, BERT can be used to classify the sentiments of posts due to its limited max length. This study leveraged the approach BERT to classify the Weibo sentiments of involution in different age groups.

This study used LDA and causation coding to identify the causal relationship between involution and group cohesion in youth’s Weibo.

Following this, this study built a STM to discover topics and estimate their relationship to document meta-data. Outputs of the model can be used to conduct hypothesis testing regarding different opinions among different generational groups. This mirrors the type of analyses social scientists perform with content analysis in small data.

Lastly, this study analyzed the hashtags used by youth to discuss involution and build group cohesion. The data corpus for this study’s analysis was public Weibo posts in which the hashtags related to involution appeared in youth posts. Data were collected and analyzed by coding and axial coding (Saldaña, 2021) was used to study the hashtags.

### 3.1. Data collection

By following a consolidated strategy, all hashtags and keywords that involved involution in collecting posts posted to a microblogging platform (Weibo) were selected to avoid irrelevant posts. The list contained various general involution issue-related terms: #involution is everywhere, #what is involution, #why we start the involution, #anti-involution, #Youth should fight against the “involution” trend, #How does the youth live if they fight against involution, #How to avoid involution, etc. The Weibo stream was monitored, and data was collected using the Weibo Search application programming interface (API); this allowed for the collection of nearly all posts containing any search terms. The Weibo topic interface was selected to ensure that all posts containing the involution of interest posted during the data collection period were obtained; this precaution avoided incurring known sampling issues related to collecting data. This procedure yielded a data set containing 20 thousand unique tweets.

### 3.2. Sentiment detection

Various strategies to label social media sentiments exist (Liu et al., 2017). With neural networks’ capacity of learning representation from data, deep learning has become one of the most useful research models in this area. Based on Long Short-Term Memory (LSTM) and memory networks, BERT rely on the attention mechanism to label the context, which unifies these two stages, as encoding a concatenated text pair with self-attention effectively includes bidirectional cross attention between two sentences. BERT performs excellently in sentence-level sentiment classification (SST-2; Devlin et al., 2018; Gao et al., 2019). Thus, BERT can be used to classify the sentiments of posts due to its limited max length (140 words). This study leveraged the BERT approach to classify the Weibo sentiments of involution in different age groups.

### 3.3. Fine-tuning model training

This study focused on fine-tuning training and used the pre-training model ‘hfl/chinese-roberta-wwm-ext’ trained by Yiming Cui, who built the Chinese pre-trained language model series and released them to the public to facilitate the research community. Fine-tuning is straightforward since the self-attention mechanism in the transformer allows BERT to model several downstream tasks—whether they involve single text or text pairs—by swapping out the appropriate inputs and outputs.

Regarding finetuning, the BERT model was first initialized with the pre-trained parameters, and all parameters were fine-tuned using labeled data. Sentiments of 3,000 Weibo including involution (0-negative, 1-positive) were labeled, and the pre-trained uncased base model of BERT2 was fine-tuned on a single NVIDIA RTX 3070Ti GPU (16GB RAM).

BERT models were trained on multiple datasets and achieved high detection accuracy (>90%) on cross-validation benchmarks. This study’s reference model was selected for its best trade-off between scalability and accuracy; the model is very precise at detecting sentiments about involution—precision rate (PR) of 96%.

After the detection, the *SIC* and Age group sentiments were calculated. Every day’s Sentiment Influence Calculation (*SIC*) of discussion posts on Weibo:

The influence of Weibo’s sentiments was calculated as follows:


$$\text{SIC}=\Sigma j\left({{\text{Likes}}_{ij}}^{*}{\text{sentiment}}_{ij}\right)$$

The identified negative and positive sentiments were calculated in different age groups as follows:


$$\text{Age}\;\text{Group Sentiments}=\Sigma j\left({\text{sentiment}}_{ij}\right)$$

### 3.4. LDA and STM

The Latent Dirichlet Allocation (LDA; Blei et al., 2003) is a generative statistical model that explains a set of observations through unobserved groups. Each group explains why some parts of the data are similar in natural language processing.

This study built a STM to discover topics and estimate their relationship to document meta-data. Building off of the tradition of probabilistic topic models, such as the Latent Dirichlet Allocation (LDA) and other topic models that have extended these (e.g., Gerrish and Blei, 2012; Paul and Dredze, 2015), the STM’s key innovation is that it permits users to incorporate arbitrary metadata, defined as information about each document, into the topic model. Outputs of the model can be used to conduct hypothesis testing regarding different opinions between different age groups. This mirrors the type of analyses that social scientists perform with content analysis.

The STM approach to estimation builds on prior work on variational inference for topic models (Blei and Lafferty, 2007). Researchers develop a partially collapsed variational Expectation–Maximization algorithm that supplies estimates of the model parameters upon convergence. Regularizing prior distributions are used for γ, κ, and (optionally), which help enhance interpretation and prevent overfitting.

Its implementation in the STM R package provides tools for machine-assisted content analysis (Roberts et al., 2019). First, the data is ingested and prepared for analysis. Following this, a structural topic model is estimated. Finally, the results are evaluated and visualized.

### 3.5. Axial coding

The goal of Axial Coding is to strategically reassemble data that were “split” or “fractured.” Landsheer and Boeije (2010) explained that Axial Coding’s purpose is “to determine which [codes] in the research are the dominant ones and which are the less important ones … [and to] reorganize the data set; synonyms are crossed out, redundant codes are removed, and the best representative codes are selected.” This method “relates categories to subcategories [and] specifies the properties and dimensions of a category” (Saldaña, 2021). Properties (i.e., characteristics or attributes) and dimensions (the location of a property along a continuum or range) of a category refer to such components as the contexts, conditions, interactions, and consequences and can indicate how youth build group cohesion online.

### 3.6. Causation coding

Causation coding is a way to label models of individuals’ words to uncover “what people believe about events and their causes. … An attribution is an expression of the way a person thinks about the relationship between a cause and an outcome,” and an attribution can consist of an event, action, or characteristic (Munton et al., 1999, pp. 5–6). Three elements are needed when analyzing causality: the cause, the outcome, and the link between the cause and the outcome (Saldaña, 2021), and the results can show how involution influences cohesion offline.

### 3.7. Ethics statement

Only publicly available data were used for the analysis (according to Weibo’s specification settings). Thus, data from users with privacy restrictions were not included in the dataset.

Further, the domains from which the data were downloaded are public Weibo entities and can be accessed by anyone.

## 4. Results

**Figure 2:**
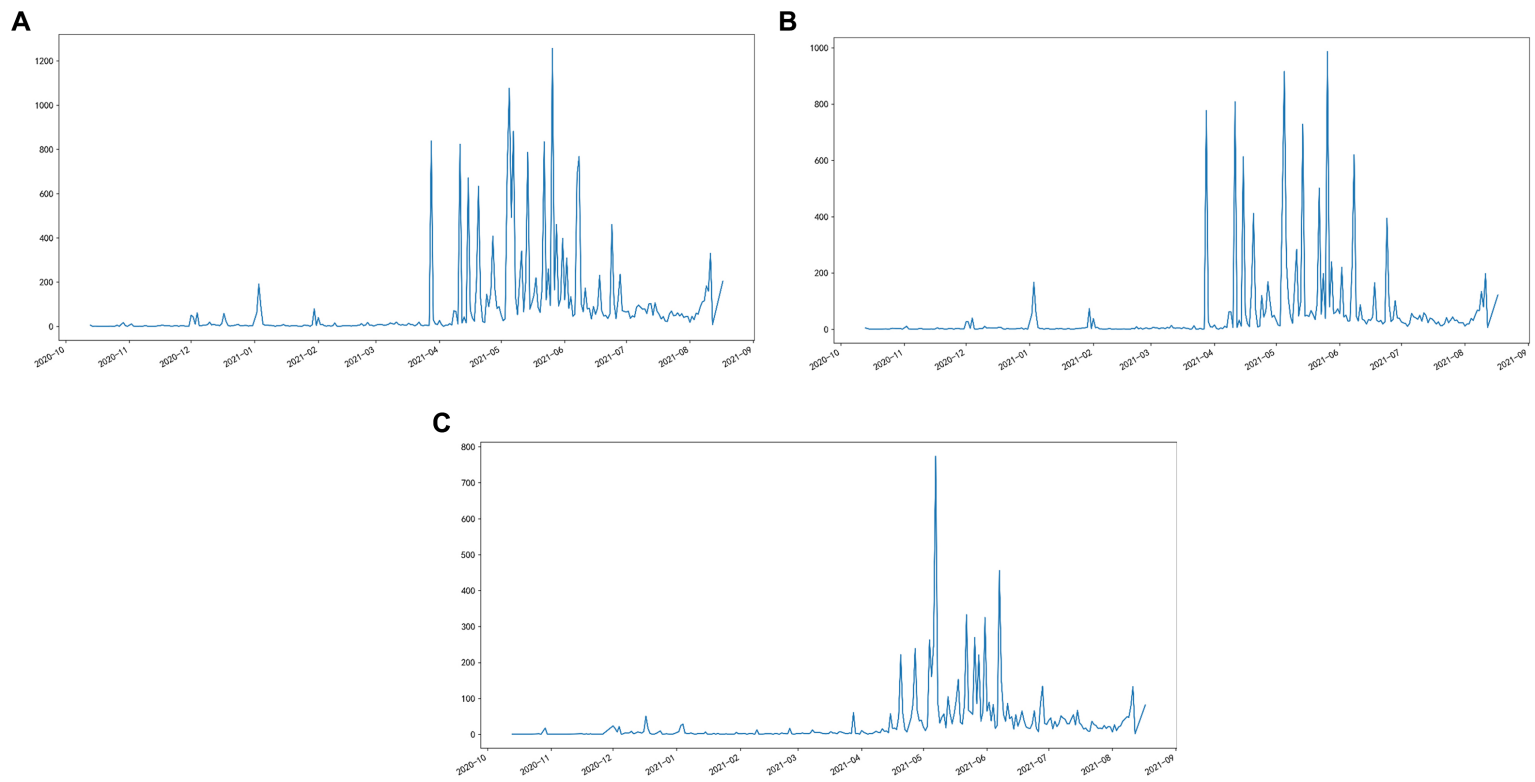
**(A)** The number of daily discussion posts on involution on Weibo. **(B)** The number of daily negative posts on involution on Weibo. **(C)** The number of daily positive posts on involution on Weibo.

Figure 2A charts the number of daily discussion posts on Weibo, indicating a clear trend. Figure 2B illustrates the number of daily negative posts on involution on Weibo. As can be seen from this figure, the involution began to erupt on Weibo in April 2021, increasing in May and June 2021, and decreasing over time. Throughout this period, the involution garnered significant negative discussions. From the user profile, many youths voiced their experiences and opinions at that time. However, positive Weibo had notably fewer posts than negative ones, and voices on the platform were relatively late to the negative Weibo (shown in Figure 2C).

### 4.1. Sentiments detection

**Figure 3:**
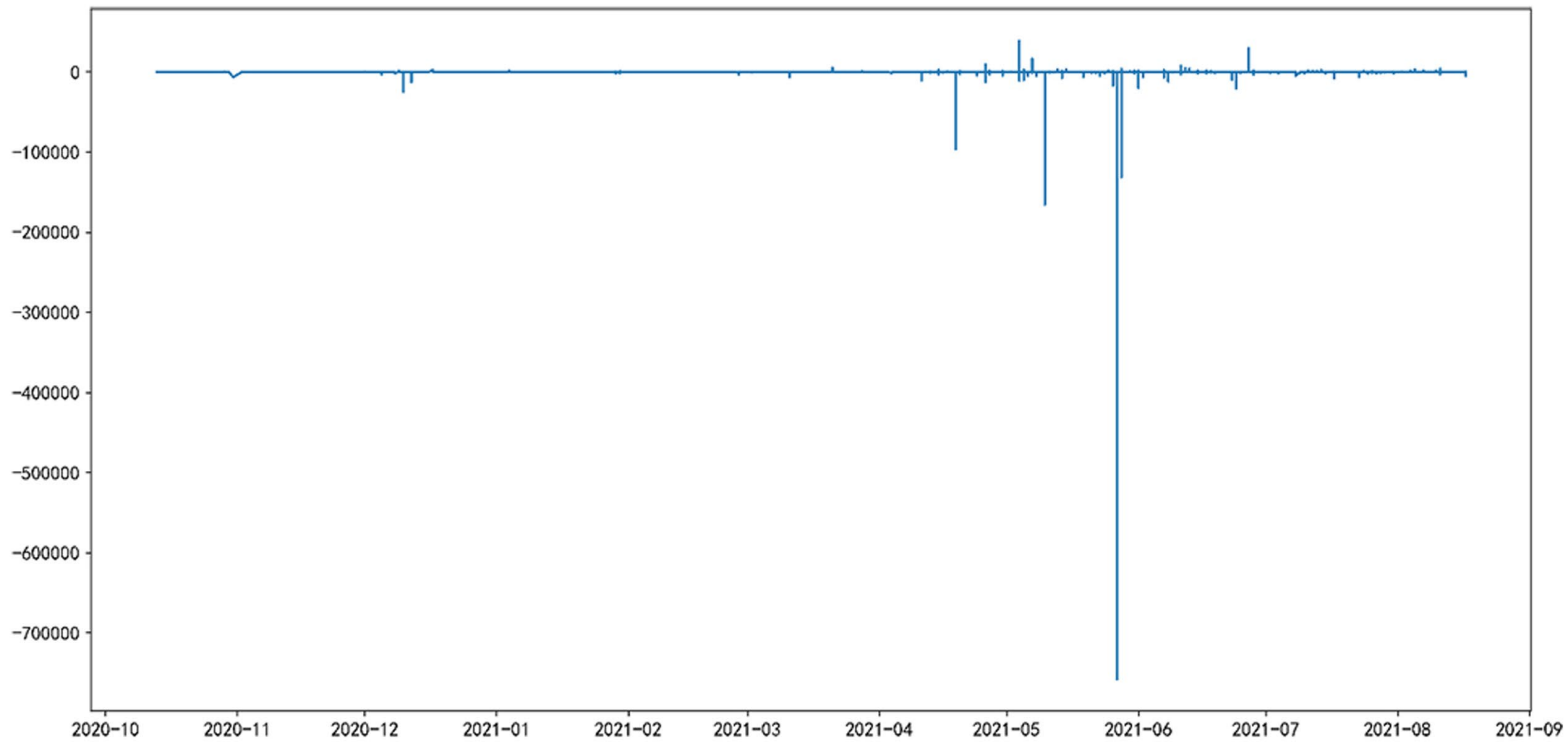
Every day’s Sentiment Influence Calculation (*SIC*) of discussion posts on Weibo.

Figure 3 presents the everyday Sentiment Influence Calculation (*SIC*) of discussion posts on Weibo. From this figure, it can be inferred that the negative posts were more influential, especially in May and June. Youths appealed to resonance and received more likes. For instance, a man aged 30 posted: “Generation Z starts to fight against the involution”^1^ and received 16 thousand likes.

China’s definitions of generations are relatively vague. Such as the “millennials” and “Generation Z,” which are often mentioned in China, also followed the international definitions of generations. Moreover, several studies used the international classification method on generations to define Chinese generations and conducted research accordingly (e.g., Long and Wang, 2015; Ao, 2021). Consequently, this study also adopted the same international generation classification method.

The definition of youth should also be discussed in the Chinese social context. Zhijian Huang, a Chinese scholar, conducted a literature review on the age limits of youth. He found the lower limit of youth age to be approximately 14 years old, and the upper limit of youth age varies, ranging from 24 to 49 years old (Long and Wang, 2015). According to the generational group used in the present study, individuals aged 16–40 were grouped as youth, including Generation Y and Z.

**Figure 4:**
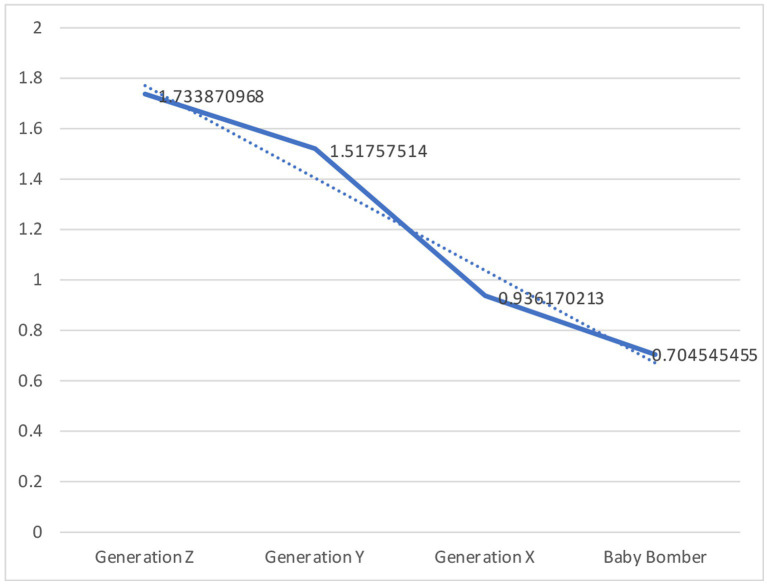
The negative/positive sentiment ratio trend in different generational groups.

Figure 4 presents the negative/positive sentiment ratio trend in the different generational groups. The users aged 16–24 (Generation Z) exhibited more negative attitudes than positive attitudes (1.73:1); the users aged 24–39 (Generation Y) exhibited more negative attitudes than positive attitudes (1.52:1); the users aged 40–54 (Generation X) exhibited fewer negative attitudes than positive attitudes (0.94:1); and the users aged over 55 (Baby Bomber) exhibited significantly fewer negative attitudes than positive attitudes (0.70:1). This indicated that a generational gap exists when discussing involution; younger individuals are more negative than older adults.

### 4.2. Involution and group cohesion offline

To understand the relationship between involution and group cohesion offline, only the youth’s Weibo was selected and the keywords “friend,” “classmates,” and “colleague,” which refer to “group member” in the involution context, and “workplace,” “company,” “schools” which refer to “group” according to the dimension Sargent and Sue-Chan proposed was searched for. First, the sentiments ratio was calculated, and it was found that the ratio of positive sentiments was relatively low. Following this, LDA was used to find the typical opinions and analyze them. The results indicated four major categories: (a) stop irrational competition with others; (b) how “involution” can you become; (c) please do not start endless competition; and (d) stop doing this to your mates. The results were so similar that a second step, manual coding, was required. Causation coding was applied to analyze the relationship between involution and group cohesion. After coding the corpus, six causal links (“I hate my colleagues because they get me into involution,” “I hate the company because it pushes me to endless competition,” “I feel no belonging to the workplace because I cannot get involved in their involution game,” “I would like to quit my job because of my stupid and aggressive colleague,” “I would like to quit my job because of the stupid involution game,” and “I felt despondent about work because of the involution”) emerged and were compared with Sargent and Sue-Chan’s (2001) dimension in the discussion.

First, it appeared that involution made the users dislike their workgroup. For example: “I was ‘involuted’ by a colleague the very first time, and he used to be a good friend… damn it, it is such a bad place.”^2^ “…This is such a disgusting company, why are colleagues so eager to work overtime…The company is horrible due to this involution.”^3^

Second, users felt that they did not belong to their peer group: “All the candidates graduated from great universities like 985, 211…and they are looking for jobs for several months…I felt I am so out of place…”. ^4^

Third, users felt sad about work because of the involution: “…I have many same experiences: the colleagues will not take responsibility and blame each other, pushing others to settle things…company is full of this kind of involution pressure. So many things are like this…It seems that we are doing many things, but actually, we are doing nothing. I felt really exhausted about such bad relationships…”.^5^

Fourth, users experienced hatred toward their colleagues because they believed that they got them into involution: “Since I got my new job and worked for the bigger company, I found my colleagues are all fools. They like involution so much. Everyone is so aggressive and fights for a low salary. I am so desperate,”^6^ “The complicated relationship between colleagues made me feel impossible to become friends with them…and the involution is everywhere…”.^7^

Besides, users were trying to quit their groups due to aggressive colleagues and involution: “Do you want to quit your job at the bank? The new colleague is so stinky and neijuan…He is so neijuan that it kills me…”.^8^

### 4.3. STM

**Figure 5:**
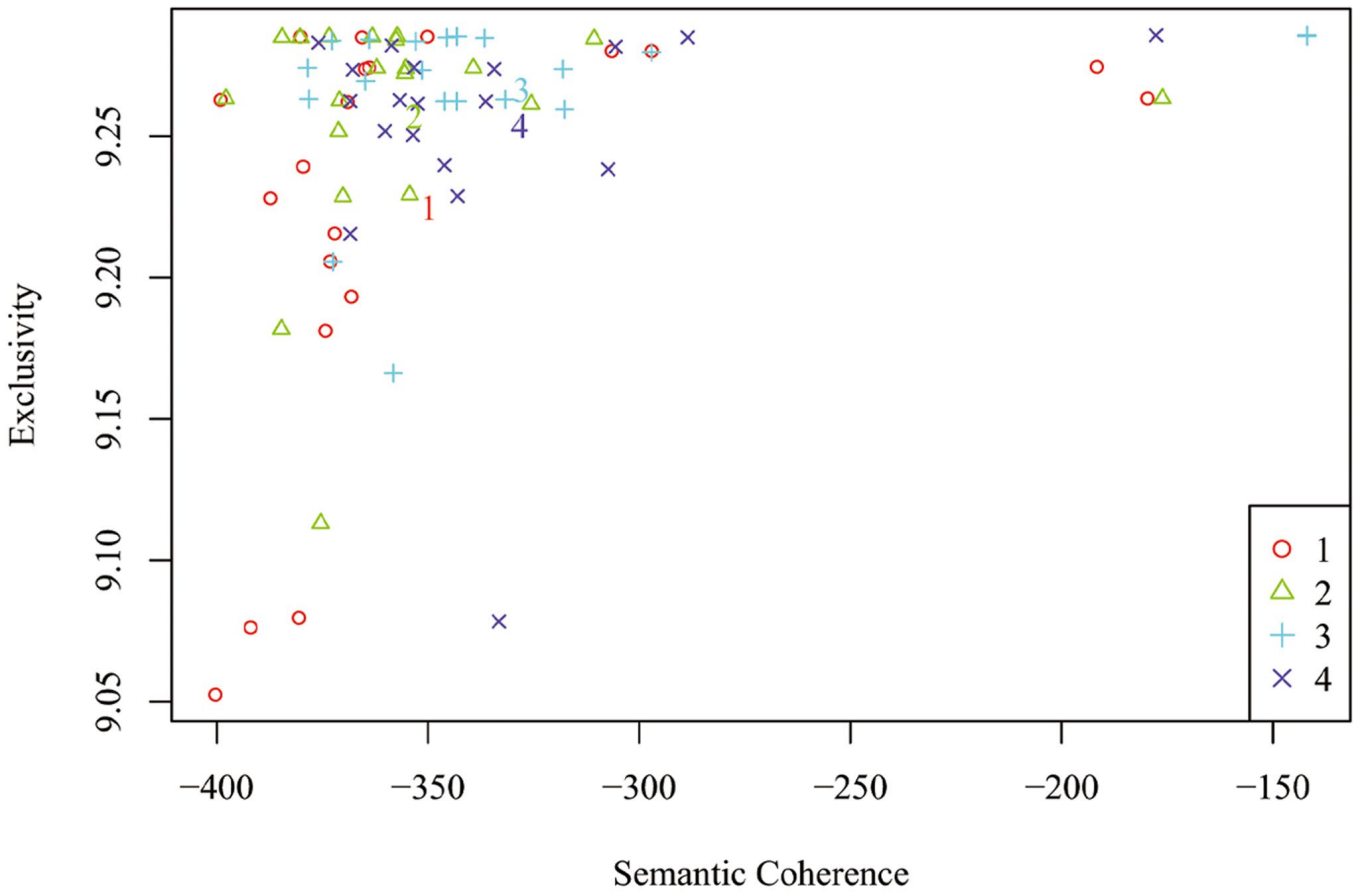
Plot of selected Model results. Numerals represent the average for each model, and dots represent topic-specific scores.

Understanding the needs of the different groups, how they think, and how they work are keys to understanding why the generation gap exists (Hill et al., 2003), and whether outgroup pressure pushes them to unite. To answer RQ2 by STM, this study used age as an indicator variable for the treatment condition and an interaction between age group and treatment condition as covariates. The interaction term allows for the examination of whether older adults respond differently to the treatment condition than youths. In this particular application, the influence of these parameters was estimated on topic proportions (“prevalence”) within responses. To address multi-modality, this study’s model was estimated 20 times, with 20 different starting values, and the model selection procedure described earlier was applied. Since these models indicated similar results regarding the topics discovered and differences in topic proportions across treatment conditions, one was selected based on exclusivity and semantic coherence criterion (shown in Figure 5).

**Figure 6:**
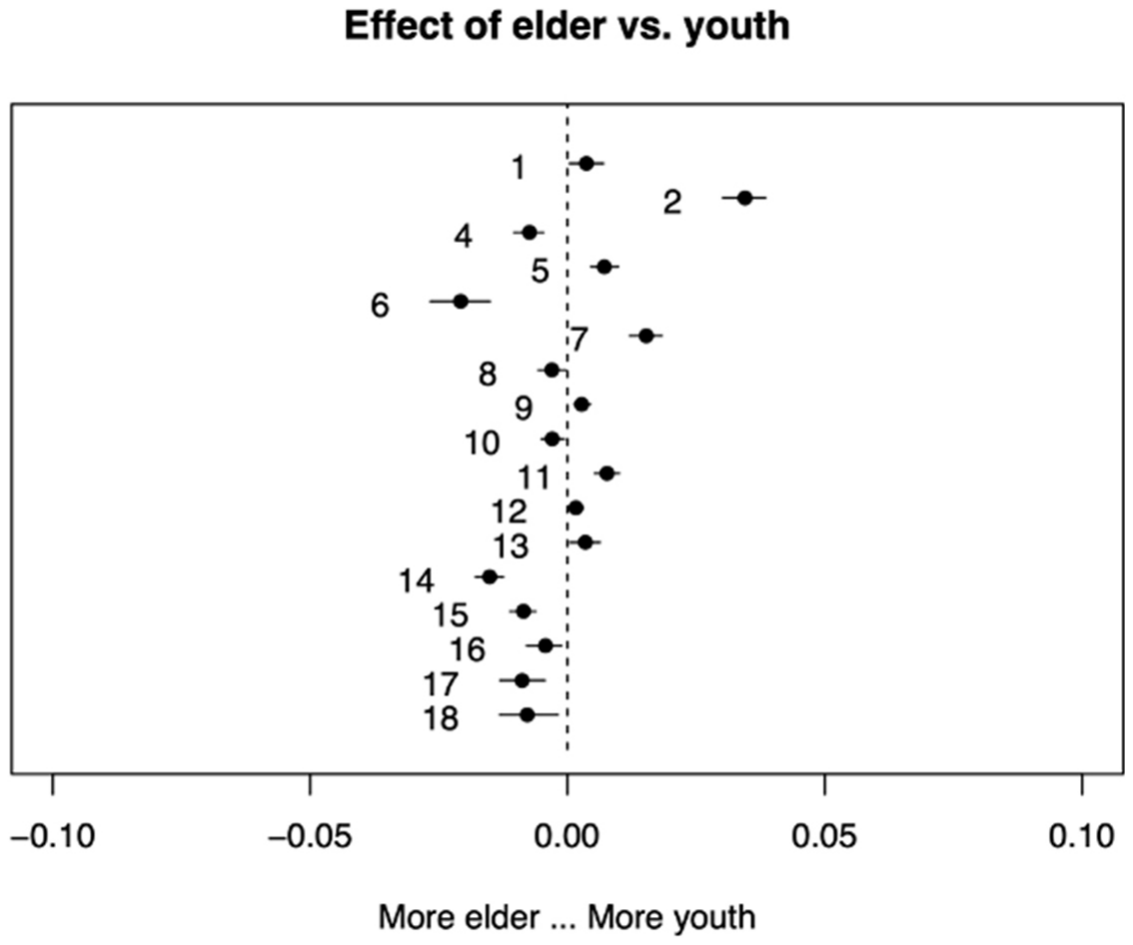
Graphical display of topical prevalence contrast.

A total of 20 topics were estimated in this study’s analysis. The 17 topics most associated with the treatment and control groups are presented as follows: Topic 1 was the “involution in the workplace” topic; Topic 2 emphasized that working hard was a way out; Topic 4 highlighted that youths should fight against involution; Topic 5 was the “overreaction to involution” topic; Topic 6 stressed that students should fight against involution; Topic 7 was the “involution is not a new phenomenon” topic; Topic 8 emphasized how to deal with involution; Topic 9 was about “involution in the workplace”; Topic 10 emphasizes appearance anxiety; Topic 11 was the “involution spread widely in our society” topic; Topic 12 emphasized the meaning of involution; Topic 13 was the “overseas students aggravate involution” topic; Topic 14 referred to the reason we should fight against involution (because it is harmful and useless). Topic 15 is the “education involution” topic; Topic 16 discussed how older adults do not understand because they are not involved (in the involution youth face); Topic 17 was the “depreciation of education level is a kind of involution” topic; and Topic 18 emphasized that involution does not mean making efforts (shown in Table 1 and Figure 6).

Following this, this study moved to differences across treatment groups. On average, across both treatment and control groups, topics 4, 6, 8, 10, 14, 15, 16, 17, and 18 were more likely to be discussed by youths, while topics 1, 2, 5, 7, 9,11, 12, and13 were discussed more by older adults. In addition, topic 2 was discussed significantly more by older adults than youths, which indicated that older adults encourage individuals to work instead of questioning. Youths discussed pressure and desperation toward involution significantly more than older adults. The results showed that youths were worried about the grinding work and learning culture, but there does not seem to be a way out, while older adults, who experienced arduous old days, might accept the competition idea to a greater extent.

**Table 1:** Topical prevalence contrast.

Topic	Content	Group category
Topic 1	Involution in the workplace	Elder
Topic 2	Working hard is a way out	Elder
Topic 4	Youths should fight against involution	Younger
Topic 5	Overreacting to involution	Elder
Topic 6	Students should fight against involution	Younger
Topic 7	Involution is not a new phenomenon	Elder
Topic 8	How to deal with involution	Younger
Topic 9	Involution in the workplace	Elder
Topic 10	Appearance anxiety	Younger
Topic 11	Involution spread widely in our society	Elder
Topic 12	The meanings of involution	Elder
Topic 13	Overseas students aggravate involution	Elder
Topic 14	Fight against involution because it is harmful and useless	Younger
Topic 15	Education involution	Younger
Topic 16	Elders do not understand because they are not involved	Younger
Topic 17	Depreciation of education level is a kind of involution	Younger
Topic 18	Involution does not mean making efforts	Younger

### 4.4. Hashtags

Based on youth Weibo posts, 369 topics related to involution were detected. Two researchers were involved in applying the initial coding frames based on the detected hashtags. First, they manually deleted hashtags unrelated to the real meaning of involution, such as “fruits are involved in involution,” after which a total of 96 topics remained. Following this, axial coding was applied to find the main categories youth use to discuss involution. Their discussions led to a final set of four major categories: (a) we are fighting against the involution; (b) the involution in daily life; (c) the explanation of involution; and (d) the strategies to fight against the involution (as shown in Table 2).

**Table 2:** The axial coding of hashtags related to involution.

Main-categories	Sub-categories
We are fighting against the involution	We refuse involution in workplace
	Youth are fighting against involution
	Fighting against the education involution
	Students are fighting against involution
	Hope we can effectively stop involution
	Upper-class elders support the involution
The involution in daily life	The involution in daily life
	The aggravation of involution in daily life
	The opinions toward involution in daily life
	People who are parenting face involution
	People who are studying face involution
	The influence of involution
	People who are working face involution
	Females are facing fierce involution
	People who are looking for a job face involution
The explanation of involution	The involution and the success
	The explanation of involution
	Celebrities’ opinions about involution
	Experts’ opinions about involution
	The reason for involution
	Straight A students’ opinions about involution
	Entrepreneurs’ opinions about involution
The strategies to fight against the involution	The strategies to fight against the involution
	The strategies to fight against the education-related involution

Coding was followed by thematic analysis. This phase aimed to analyze the coding frame in the context of previous studies on the generational gap and group cohesion. Applying these concepts meant looking at each frame’s discursive and relational dimensions. Thus, each frame was thematically analyzed based on what resources contributors employed to participate in the discussion and how they positioned themselves with ‘the others.’ Each frame was analyzed separately, and two themes (‘revealing social identity,’ ‘becoming and positioning’) emerged and were compared across frames. The final interpretation of themes resulted from a back-and-forth between the literature on group cohesion, individual and joined data analysis, and interpretation.

These hashtags made youth share their experiences, support each other, and fight against the odds as a group. These findings showed how online group cohesion could be strengthened through hashtags.

## 5. Discussion

Studying involution on Weibo might be a way to understand why youth strengthen group cohesion to mitigate the crises, whether it be fierce competition in the workplace or increased tensions in schools, and help youth face these difficulties. First, LDA and causation coding was used to examine whether there were causal links between involution and group cohesion. Following this, this study’s empirical strategy enabled the analysis of the attitudes of the age group toward involution and examined opinion differences between youth and older adults. Strong evidence supporting the existence of a generation gap was found through the BERT techniques in detecting sentiments and STM methods to analyze contents from a large-scale discussion of involution. Harnessing the sentiment and the semantic content of the Weibo dataset, this study identified that different age groups had opposite stances. The results showed that youths were more negative than older adults. After content analysis using STM, it was found that youths were more likely to feel pressured and trapped in endless and useless competition games and blamed their colleagues for getting them involved in involution. In contrast, older adults were more likely to accept the competition game. Lastly, this study explored what hashtags youths used to gain attention, appeal to resonance, and strengthen their identity-based cohesion.

The results of RQ1 showed that involution caused poor group cohesion and youth stressed their poor group cohesion due to irrational involution. All the LDA results were similar, showing that individuals were complaining to their colleagues and calling for the stop of involution. The causation coding results showed a clear trend suggesting that involution caused bad group cohesion outcomes. According to Sargent and Sue-Chan’s dimension (2001), poor group cohesion was shown through the users’ Weibo. First, they hated their group due to involution. Second, they expressed their pressure and unwillingness to work. Third, they felt exhausted due to the endless competition and even wanted to quit their jobs. Moreover, they felt like they did not belong to their work group because of involution.

Furthermore, they hated their group members due to the involution, let alone building a good relationship with them. Involution caused colleagues to be involved in an endless and useless game at work. If youth want to ‘survive,’ they must outcompete others, even their friends. This is in line with Lyles’s view (2018) that friendly competition and cooperation were consistently strong predictors of cohesion. Thus, involution, the endless and useless competition, damages the friendships between colleagues and triggers poor group cohesion.

Regarding the generation gap and identity-based group cohesion on social media. This study found that a generation gap exists from the results of RQ2. As stated by Hill et al. (2003), understanding the needs of the different groups, how they think, and how they work are keys to understanding why the generation gap exists. To answer RQ3, this study analyzed the attitudes and opinions of different generations to identify how they unite and why inter-group conflict exists. Additionally, this study focused on the social identity-related words in the different groups shown in STM, since it is crucial to group cohesion (Lee et al., 2020). The results indicated two reasons that explain online group cohesion.

The first reason why youth united online was due to their different life experiences compared to older adults. The older adults, the ones born before 1965 and known as “Baby Boomers,” had positive attitudes toward involution. Scholars describe this generation as workaholics who rarely job-hop (Angeline, 2011). In China, they are leaders and authorities in several areas and experienced difficult times and wartime when they were young (Sun and Cheng, 2018). Gen X employees, aged 40–55, according to Gursoy et al. (2008), are efficient problem solvers but choose not to take on additional work where possible. Thus, their attitudes are not as positive as the older group’s when facing endless competition. In conclusion, the polarized opinions showed that the older adult group tended to believe that “involution is not a new phenomenon” and that youth were “overreacting to involution” because they were hard workers themselves when they were young. While the youths consisted of individuals aged 18–40, that is, most of them are generation Y and Z. Generation Y employees, aged 24–40, are casual, expect managers to know them by their names, understand their needs and expectations to promote, and care for their well-being and own achievement rather than the collective goal (Gursoy et al., 2008). As Generation Z grew up in a competitive environment, a survey conducted by Half (2015) indicated that approximately 80% of the members of Generation Z expect to work harder to have a successful professional path, which is against the useless and endless competition games that cannot create promotion opportunities. Thus, it is plausible that their opinion is “fight against involution because it is harmful and useless.” Not restricted to the workplace, youth who are students discussed their involution in school, including graduation and looking for a job. The polarized opinions “depreciation of education level is a kind of involution” and “overseas students aggravate involution” can explain why youth united online and voiced their opinions. The “students should fight against involution” opinion indicated that they support each other and attempt to find a way out together.

When youth and older adults start to voice their opinions, they will find each other ridiculous. A professor supports the involution by explaining its meaning: “People are talking about involution, which shows that society can gain opportunities to rise through competition. Otherwise, the social stratum will be solidified, all efforts will be in vain, and society may face great turbulence and regression.” Thousands of youths started to leave hate comments on this news and started to self-categorize to create group cohesion. Thus, compared to the older group who express their opinions, the STM results showed that youth are more likely to use identity-related words to express their opinions (e.g., “youths should fight against involution,” “overseas students aggravate involution”), which indicated a group cohesion trend. Labov (2010) explained that language is a key marker in a socially constructed experience of identity formation. Thus, youth’s identity-related expressions are based on the development of group construction (Lee and Toutanova, 2018) and thus facilitate group cohesion.

Second, youth try to find the root of the irrational involution from a social structure perspective. From this perspective, youth stress their collective identity by blaming “others.” They start to blame the older group with opinions such as “older adults do not understand because they are not involved.” Youths struggle to survive, and they find that older adults, such as leaders and bosses, especially in the workplace, live easy lives. Thus, they are more likely to attack older adults who show support for involution. Once celebrities, especially successful older celebrities, support involution, they receive endless hate speech. The different positions refer to the unity of components, which is the difference between “other” and “us.” In addition, when young individuals regard successful older adults as “others,” they compare and even begin to complain, believing that the older adults are the ones to be blamed for their continued involution and the ones who obtain benefits from involution. For example, a youth wrote this post in an online involution discussion: “…I am speechless. In the workplace, I was passive. They (the capitalists) had high daily salaries but were not satisfied. I worked 996^9^ a week and they thought I was not hard-working and lazy. It was the capitalists who tricked us”^10^ Through this process, young individuals began to group, and opinions developed in an increasingly polarized direction. Regarding ICT, youths rely heavily on social networking facilities such as Weibo and Twitter to communicate and complain. Some older adults discuss their confusion about involution online. Consequently, when discussing involution online, the generation gap appears, and youth start to unite.

To conclude the results of RQ2 and RQ3, the gap regarding competition and promotion way at work from different generations was identified as one of the primary sources of inter-generational diversity understandings of involution. Youth start to stress their in-group values and understanding of competition. Another main reason is the different positions youth and older adults are in when facing involution.

From the results of RQ4, the hashtags used by youth to appeal to resonance had two main mechanisms. First, various identities were shown in the hashtags to appeal to more identity-related group members to join. Hashtags seen in category (b) “The involution in daily life” included “youth who are generation Z start to get involved in involution,” “college students are in fierce involution” and “how could a 35-Year-old employee get out of involution.” With the focus on one identity, individuals shared their bad experiences and made age groups directly related to involution using hashtags. Users with the same social identities categorized themselves and joined this hashtag trend. Since social identity arises when an individual shares interests with his or her social group (Rousseau, 1989; Meyer et al., 2006), this self-categorized procedure formulates group cohesion. Moreover, the age-related hashtags appeared in category (a) “We are fighting against the involution.” Personal stories were shared on these topics and were open-ended enough to allow users to customize their narratives (Clark, 2016). For instance, Weibo user “双门洞德善nei” wrote, “It is too difficult…colleagues start to go off work at 10 pm…and others said it is amazing to just leave at 10 pm. I could say nothing but awesome.” with the hashtag #Youth are fighting against the involution. These categories highlighted the centrality of victims’ stories for the construction of the identity of a battered youth in this unfair game. “I’ve been Singapore for three years…I’ve been leaving ‘neijuan’ for such a long time and now I felt less anxious about life…” Aziz (2022) argues, reclaim identity narratives through visibility social media are crucial to the formation of social groupings, facilitated identity construction. In these ways, the youth strengthen group cohesion.

“Positioning and Becoming” are mechanisms youth use to spread the influence of hashtags. Hashtags such as “please explain the involution from your major,” “did the involution appear in your life?” and “how to fight against the involution in your life” appeared in categories (c) “The explanation of involution,” and (d) “The strategies to fight against involution.” All these hashtags caused youth who did not consider the involution to reevaluate and position themselves as victims. For instance, Weibo user “zmxs-小黑小白” wrote, “Today, I learned that my classmates also thought a lot when they were looking for a new job. More than one of them chose to resign one year after graduation. This reality is different from the world we thought when we graduated. At that time, I was full of ambition, but later I gradually lost my way; now I realize that I became a small gear in this super volume era. We naively believe that the only way to escape is to jump from a small pit to a slightly larger pit, where we can beg to find a small sky. I am starting to get confused” with the hashtag “How to treat the involution phenomena.” As Cappellini et al. (2019) claimed, hashtags have a constitutive role; particularly when concerned with social issues, they can be conceived as sites for articulation, adaptation, and contestations of ideas, identities, and non/belonging. They are “both markers of belonging and places of becoming” (Brambilla, 2015).

By attaching a hashtag to one’s content, the youth acts to make their content more visible to others; it is an intentional act designed to create affiliation (Foster et al., 2021). The “revealing social identity” and “positioning and becoming” hashtag strategies helped create an online space for youth with different experiences and memories to engage with one another. Thus, consistent with dynamic models of social identity theory (Becker and Tausch, 2015; Drury et al., 2016), participating in hashtags may serve to strengthen social identity (Rosenbaum, 2019). In turn, this group identification process is fundamental for creating solidarity (Meyer et al., 2006; Harlow and Benbrook, 2019). Thus, given the established relationship based on social identity-related groups (e.g., Foster et al., 2021), Weibo users represent themselves as victims who struggle to survive and unite online to find a way out from the fierce and useless competition they face.

To conclude, when a collection of individuals perceives themselves as a group, a construct known as entitativity (Campbell, 1958), psychological and interpersonal changes occur (Harasty, 1996; Forsyth and Elliott, 1999). Compared to poor group cohesion offline, they use social media to build identity-based group cohesion to fight against involution. Youths might hope that online group cohesion affords structural conditions to produce collective identity and group participation (Lee et al., 2020). These new social ties, which refer to the emotional connection among youth online, are ways to alleviate youth competition pressure when facing peers offline. By stressing the structural roots, youth discuss how to combat irrational involution, return to rational competition.

These findings should raise awareness from the government because the processes set alarms to the crisis of social stagnancy. Helping youth develop themselves efficiently is one method of stopping the crisis. From a practical perspective, the Chinese government should rethink the youth’s online group cohesion and find ways to solve the plight of young individuals’ blocked promotion channels and lack of job opportunities. The government has begun to control house prices, however, much more should be done. For instance, it is necessary to create more job opportunities. Given the “996” fierce working schedule and “35-year-old workplace crisis” related posts, the government should introduce more effective policies requiring enterprises to guarantee young individuals’ spare time after work. Additionally, the government should focus on opinion polarization which may cause more conflicts. Furthermore, the government and media should focus on correcting each generation’s misperceptions of others, and leaders in the workplace and on campus should create teams to enable youth to collaborate rather than compete with their peers and seniors.

## 6. Contributions, limitations, and future work

By employing the concept of the generational gap in involution, this study advances the understanding of group cohesion in three ways: first, this study discussed one of the most important crises, involution. Dissimilar to other crises, involution means irrational competition in a group, which also closely relates to group cohesion. To the best of our knowledge, few previous group cohesion-related studies focused on this critical concept. This study identified the relationship between involution and poor group cohesion. Additionally, how to combat irrational involution, return rational competition to its roots, and eliminate irrational competition is a research field that requires attention. This study explained how young individuals voice their opinions on social media and build internal cohesion. Second, this study indicated that online opinion polarization between different identity-based groups can be seen as a way to examine the existence of group cohesion. Intergroup conflict may assist opposing groups in more clearly identifying their respective group’s enemy and thus provide a concrete basis for identity formation and increased identification with the group (Turner et al., 1979). Third, this study indicated how hashtags, regarded as bordering spaces (Cappellini et al., 2019), can strengthen group cohesion through mechanisms related to social identity theory when concerned with hot issues. Specifically, this study proposed a new combined computational method to understand group cohesion and involution.

This study has some limitations and thus facilitates future research. First, compared to psychological groups (i.e., less aggressive members, workaholics), this study only focused on the social-identity-based group. This was because involution has a social structure root, so finding the groups on Weibo might be easier, particularly when dealing with large online datasets. Future studies should consider different psychological groups’ opinions toward involution with a small sample so that more information about participants can be gained. Second, future studies should focus on whether online identity-based group cohesion facilitates offline social connectivity. Additionally, whether online group cohesion will recreate a productive workforce needs to be considered. Third, we only retrieved revolution-related posts on Weibo as the basis of the textual analysis. However, the occurrence of social events in this interval may cause differences in group views, leading to biased results because of the spiral of silence. Many other methods, like social networking, questionnaire, or in-depth interview, can be applied for further study. Since Many Chinese hot issue studies are based on content on Weibo, so we chose Weibo platform to extract data. Future studies can focus on other platforms, and of course, other countries’ social media platforms.

## Data availability statement

The original contributions presented in the study are included in the article/supplementary material, further inquiries can be directed to the corresponding author.

## Author contributions

YZ designed this study, performed the data analysis, and wrote the manuscript. TJ did the literature review, coded, and wrote part of the article. All authors approved the submitted version.

## Conflict of interest

The authors declare that the research was conducted in the absence of any commercial or financial relationships that could be construed as a potential conflict of interest.

## Publisher’s note

All claims expressed in this article are solely those of the authors and do not necessarily represent those of their affiliated organizations, or those of the publisher, the editors and the reviewers. Any product that may be evaluated in this article, or claim that may be made by its manufacturer, is not guaranteed or endorsed by the publisher.

## Footnotes

^1^外卖小哥金城武. (May 05, 2021). Retrieved from: https://weibo.com/1930072831/KePBw9dTj?refer_flag=1001030103_ (Accessed October 22, 2021).

^2^(August 21, 2021) #Retrieved from: https://weibo.com/5456542348/KvTCkleqD?refer_flag=1001030103_(Accessed October 22, 2021).

^3^(April 18, 2021) #Retrieved from: https://weibo.com/6573788020/KbvpOFb1X?refer_flag=1001030103_ (Accessed October 22, 2021).

^4^(April 28, 2021) #Retrieved from: https://weibo.com/1197152795/KcUYmz1r1?refer_flag=1001030103_(Accessed October 22, 2021).

^5^(August 31, 2021) #Retrieved from: https://weibo.com/7280838652/Kw1k0jizE?refer_flag=1001030103_(Accessed October 22, 2021).

^6^(August 31, 2021) #Retrieved from: https://weibo.com/5279855993/KvTmU7tCx?refer_flag=1001030103_(Accessed October 22, 2021).

^7^(June 24, 2021) #Retrieved from: https://weibo.com/5929956521/KlH73Ciw0?refer_flag=1001030103_(Accessed October 22, 2021).

^8^(August 07, 2021) #Retrieved from: https://weibo.com/2307804947/KsjPaELCz?refer_flag=1001030103_(Accessed October 22, 2021).

^9^“996” is a description of the fierce working schedule in China. It means that workers go to work at 9 am, get off work at 9 pm, and work six days per week.

^10^(May 5, 2021) retrieved from: https://weibo.com/6324839905/Ke3cAmTic?refer_flag=1001030103_

## References

[ref1] Angeline T. (2011). Managing generational diversity at the workplace: expectations and perceptions of different generations of employees. Afr. J. Bus. Manag. 5, 249–255. doi: 10.5897/AJBM10.335

[ref2] Ao C. B. (2021). Generation Z’s characteristics of consumption. Chinese Youth Study 6, 100–106. doi: 10.19633/j.cnki.11-2579/d.2021.0092

[ref3] Aziz A. (2022). Rohingya diaspora online: mapping the spaces of visibility, resistance and transnational identity on social media. New Media Soc.:146144482211322. doi: 10.1177/14614448221132241

[ref4] Becker J. C. Tausch N. (2015). A dynamic model of engagement in normative and non-normative collective action: psychological antecedents, consequences, and barriers. Eur. Rev. Soc. Psychol. 26, 43–92. doi: 10.1080/10463283.2015.1094265

[ref5] Bengtson V. L. (1970). The generation gap: a review and typology of social-psychological perspectives. Youth Soc. 2, 7–32. doi: 10.1177/0044118X7000200102

[ref6] Blei D. M. Lafferty J. D. (2007). A correlated topic model of science. Ann. Appl. Stat. 1, 17–35. doi: 10.1214/07-AOAS114

[ref7] Blei D. M. Ng A. Y. Jordan M. I. (2003). Latent dirichlet allocation. J. Mach. Learn. Res. 3, 993–1022.

[ref8] Bliuc A.-M. Betts J. M. Faulkner N. Vergani M. Chow R. J. Iqbal M.. (2020). The effects of local socio-political events on group cohesion in online far-right communities. PLoS One 15:e0230302. doi: 10.1371/journal.pone.0230302, PMID: 32226045 PMC7105128

[ref9] Bliuc A. M. McGarty C. Thomas E. F. Lala G. Berndsen M. Misajon R. (2015). Public division about climate change rooted in conflicting socio-political identities. Nat. Clim. Chang. 5, 226–229. doi: 10.1038/nclimate2507

[ref10] Brambilla C. (2015). Exploring the critical potential of the borderscapes concept. Geopolitics 20, 14–34. doi: 10.1080/14650045.2014.884561

[ref11] Bruns A. Highfield T. Burgess J. (2013). The Arab Spring and social media audiences: English and Arabic Twitter users and their networks. Am. Behav. Sci. 57, 871–898.

[ref12] Bump P. (2016). Political polarization is getting worse. Everywhere. The Washington Post. Available at: https://www.washingtonpost.com/news/the-fix/wp/2016/04/09/polarization-is-getting-worse-in-every-part-of-politics (Accessed October 21, 2021).

[ref13] Campbell D. T. (1958). Common fate, similarity, and other indices of the status of aggregates of persons as social entities. Behav. Sci. 3, 14–25. doi: 10.1002/bs.3830030103

[ref14] Cappellini B. Kravets O. Reppel A. (2019). Shouting on social media? A borderscapes perspective on a contentious hashtag. Technol. Forecast. Soc. Chang. 145, 428–437. doi: 10.1016/j.techfore.2018.07.016

[ref15] Cennamo L. Gardner D. (2008). Generational differences in work values, outcomes and person-organisation values fit. J. Manag. Psychol. 23, 891–906. doi: 10.1108/02683940810904385

[ref16] Clark R. (2016). “Hope in a hashtag”: the discursive activism of #WhyIStayed. Fem. Media Stud. 16, 788–804. doi: 10.1080/14680777.2016.1138235, PMID: 37014711

[ref17] Cramm J. M. Van Dijk H. M. Nieboer A. P. (2013). The importance of neighborhood social cohesion and social capital for the well being of older adults in the community. Gerontologist 53, 142–152. doi: 10.1093/geront/gns052, PMID: 22547088

[ref600] Devlin J. Chang M. W. Lee K. Toutanova K. (2018). Bert: Pre-training of deep bidirectional transformers for language understanding. arXiv. arXiv:1810.04805 [Preprint].

[ref18] Dragiewicz M. Burgess J. (2016). Domestic violence on# qanda: the “Man” question in live Twitter discussion on the Australian Broadcasting Corporation’s Q&A. Can. J. Women Law 28, 211–229. doi: 10.3138/cjwl.28.1.211, PMID: 37131181

[ref19] Drury J. Brown R. González R. Miranda D. (2016). Emergent social identity and observing social support predict social support provided by survivors in a disaster: solidarity in the 2010 Chile earthquake. Eur. J. Soc. Psychol. 46, 209–223. doi: 10.1002/ejsp.2146

[ref20] Duara P. (1988). Culture, power, and the state: rural North China, 1900–1942. Stanford: Stanford University Press. p 78.

[ref21] Duncan D. F. (1978). Measuring the generation gap: attitudes toward parents and other adults. Adolescence 13:77. 676844

[ref22] Eber N. François A. Weill L. (2021). Gender, age, and attitude toward competition. J. Econ. Behav. Organ. 192, 668–690. doi: 10.1016/j.jebo.2021.10.022, PMID: 32919064

[ref23] Festinger L. (1950). Informal social communication. Psychol. Rev. 57, 271–282. doi: 10.1037/h0056932, PMID: 14776174

[ref24] Forsyth D. R. Elliott T. R. (1999). “Group dynamics and psychological well-being: the impact of groups on adjustment and dysfunction” in The social psychology of emotional and behavioral problems: interfaces of social and clinical psychology. eds. Kowalski R. Leary M. R. (Washington, DC: American Psychological Association), 339–361.

[ref25] Forsyth S. Kolenda P. M. (1966). Competition, cooperation, and group cohesion in the ballet company. Psychiatry 29, 123–145. doi: 10.1080/00332747.1966.11023458, PMID: 5943417

[ref26] Foster M. D. Tassone A. Matheson K. (2021). Tweeting about sexism motivates further activism: a social identity perspective. Br. J. Soc. Psychol. 60, 741–764. doi: 10.1111/bjso.12431, PMID: 33283289

[ref27] Gao Z. Feng A. Song X. Wu X. (2019). Target-dependent sentiment classification with BERT. IEEE Access. 7, 154290–154299. doi: 10.1109/ACCESS.2019.2946594

[ref28] Geertz C. (1963). Agricultural involution: the process of ecological change in Indonesia. Berkeley, CA: Published for the Association of Asian Studies by University of California Press.

[ref29] Gerrish S. Blei D. (2012). How they vote: issue-adjusted models of legislative behavior. Adv. Neural Inf. Proces. Syst. 25, 2753–2761.

[ref30] Ghiocanu Ş. C. (2016). The evolution or involution of policies to combat youth unemployment? Roman. Rev. Soc. Sci. 11.

[ref31] Gillespie T. Boczkowski P. J. Foot K. A. (Eds.). (2014). Media technologies: essays on communication, materiality, and society. Cambridge: MIT Press.

[ref32] Gursoy D. Maier T. A. Chi C. G. (2008). Generational differences: an examination of work values and generational gaps in the hospitality workforce. Int. J. Hosp. Manag. 27, 448–458. doi: 10.1016/j.ijhm.2007.11.002

[ref33] Haboddin M. (2012). Menguatnya politik identitas di ranah lokal. Jurnal Studi Pemerintahan 3, 109–126. doi: 10.18196/jgp.2012.0007

[ref34] Half R. (2015). Get ready for generation Z. Available at: https://www.roberthalf.com/sites/default/files/Media_Root/images/rh-pdfs/rh_0715_wp_genz_nam_eng_sec.pdf (Accessed October 13, 2021).

[ref35] Harasty A. S. (1996). Perceiving groups as entities: the role of “entitativity” for impression formation processes and stereotype use. Columbus, OH: The Ohio State University.

[ref36] Harlow S. Benbrook A. (2019). How# Blacklivesmatter: exploring the role of hip-hop celebrities in constructing racial identity on black twitter. Inf. Commun. Soc. 22, 352–368. doi: 10.1080/1369118X.2017.1386705

[ref37] Hill R. P. Stephens D. Smith I. (2003). Corporate social responsibility: an examination of individual firm behavior. Bus. Soc. Rev. 108, 339–364. doi: 10.1111/1467-8594.00168

[ref38] Hui Y. (2009). The (un)changing world of peasants: two perspectives. J. Soc. Issues Southeast Asia 24, 18–31.

[ref39] Kang L. Jin Y. (2020). A review of involution and its psychological interpretation. Filozofia Publiczna i Edukacja Demokratyczna 9, 7–28. doi: 10.14746/fped.2020.9.1.1

[ref40] Kawachi I. Berkman L. (2000). “Social cohesion, social capital and health” in Social epidemiology. eds. Berkman L. F. Kawachi I. (New York: Oxford University Press)

[ref41] Kupperschmidt B. R. (2000). Multigeneration employees: strategies for effective management. Health Care Manag. 19, 65–76. doi: 10.1097/00126450-200019010-00011, PMID: 11183655

[ref42] Labov W. (2010). “Oral narratives of personal experience” in Cambridge encyclopedia of the language sciences. ed. Hogan P. (Cambridge: Cambridge University Press), 546–548.

[ref43] Lai M. Bosco C. Patti V. Virone D. (2015). “Debate on political reforms in twitter: a hashtag-driven analysis of political polarization” in IEEE International Conference on Data Science and Advanced Analytics (DSAA). Paris, France; IEEE; 1–9.

[ref44] Landsheer J. A. Boeije H. R. (2010). In search of content validity: facet analysis as a qualitative method to improve questionnaire design: an application in health research. Qual. Quant. 44, 59–69. doi: 10.1007/s11135-008-9179-6

[ref45] Lee S. H. Tak J. Y. Kwak E. J. Lim T. Y. (2020). Fandom, social media, and identity work: the emergence of virtual community through the pronoun “we”. Psychol. Popular Media 9:436. doi: 10.1037/ppm0000259

[ref46] Lee J. D. M. C. K. Toutanova K. (2018). Pre-training of deep bidirectional transformers for language understanding. arXiv preprint arXiv:1810.04805.

[ref47] Lindquist J. Xiang B. (2019). Space of mediation: labour migration, intermediaries and the state in Indonesia and China since the nineteenth century. Rev. Eur. Migr. Int. 35, 39–62. doi: 10.4000/remi.12529

[ref48] Liu Y. Bi J. W. Fan Z. P. (2017). A method for multi-class sentiment classification based on an improved one-vs-one (OVO) strategy and the support vector machine (SVM) algorithm. Inf. Sci. 394-395, 38–52. doi: 10.1016/j.ins.2017.02.016

[ref49] Liu S. D. Qiu Z. Q. (2004). The discussion of the concept of involution. Soc. Stud. 5, 96–110.

[ref50] Long Y. Wang L. (2015). Who is the youth? Generation Y in Chinese context. Chinese Youth Soc. Sci. Study 4, 11–16. doi: 10.16034/j.cnki.10-1318/c.2015.04.003

[ref51] Lyles A. A. Loomis C. Mama S. K. Siddiqi S. Lee R. E. (2018). Longitudinal analysis of virtual community perceptions of cohesion: the role of cooperation, communication, and competition. J. Health Psychol. 23, 1677–1688. doi: 10.1177/1359105316667794, PMID: 27630255

[ref52] Meyer J. Becker T. Van Dick R. (2006). Social identities and commitments at work: toward an integrative model. J. Organ. Behav. 27, 665–683. doi: 10.1002/job.383, PMID: 21448029

[ref53] Munton D. Soroos M. Nikitina E. Levy M. A. (1999). “Acid rain in Europe and North America” in The effectiveness of international environmental regimes. Causal connections and behavioral mechanisms. ed. Young O. R. (Cambridge, MA: MIT Press), 155–247.

[ref54] Netzer L. Gutentag T. Kim M. Y. Solak N. Tamir M. (2018). Evaluations of emotions: distinguishing between affective, behavioral and cognitive components. Personal. Individ. Differ. 135, 13–24. doi: 10.1016/j.paid.2018.06.038, PMID: 36518564

[ref55] Neugarten B. L. (1970). The old and the young in modern societies. Am. Behav. Sci. 14, 13–24. doi: 10.1177/000276427001400103

[ref56] Newson M. White F. Whitehouse H. (2022). Does loving a group mean hating its rivals? Exploring the relationship between ingroup cohesion and outgroup hostility among soccer fans. Int. J. Sport Exerc. Psychol., 1–19. doi: 10.1080/1612197X.2022.2084140

[ref57] Papacharissi Z. de Fatima Oliveira M. (2012). Affective news and networked publics: the rhythms of news storytelling on# Egypt. J. Commun. 62, 266–282. doi: 10.1111/j.1460-2466.2012.01630.x

[ref58] Pasquale F. (2015). The black box society: the secret algorithms that control money and information. Cambridge, MA: Harvard University Press.

[ref59] Paul M. Dredze M. (2015). SPRITE: generalizing topic models with structured priors. Trans. Assoc. Comput. Linguist. 3, 43–57. doi: 10.1162/tacl_a_00121

[ref60] Portwood-Stacer L. Berridge S. (2014). Introduction: privilege and difference in (online) feminist activism. Fem. Media Stud. 14, 519–520. doi: 10.1080/14680777.2014.909158

[ref61] Roberts M. E. Stewart B. M. Tingley D. (2019). Stm: an R package for structural topic models. J. Stat. Softw. 91, 1–40. doi: 10.18637/jss.v091.i02

[ref62] Rodgers K. Scobie W. (2015). Sealfies, seals and celebs: expressions of Inuit resilience in the twitter era. Interface 7, 70–97.

[ref63] Rosenbaum J. E. (2019). Degrees of freedom: exploring agency, narratives, and technological affordances in the# TakeAKnee controversy. Social Media Society 5:205630511982612. doi: 10.1177/2056305119826125

[ref64] Rousseau D. M. (1989). Psychological and implied contracts in organizations. Empl. Responsib. Rights J. 2, 121–139. doi: 10.1007/BF01384942, PMID: 36154339

[ref65] Saldaña J. (2021). The coding manual for qualitative researchers. Thousand Oaks, CA: SAGE Publications Limited. 1–440.

[ref66] Santosa M. A. Khatarina J. Suwana A. S. (2013). The progress on governing REDD+ in Indonesia. Int. J. Regional Rural Remote Law Policy, 1–17. doi: 10.5130/ijrlp.i1.2013.3356

[ref67] Sargent L. D. Sue-Chan C. (2001). Does diversity affect group efficacy? The intervening role of cohesion and task interdependence. Small Group Res. 32, 426–450. doi: 10.1177/104649640103200403

[ref68] Schmid K. Muldoon O. T. (2015). Perceived threat, social identification, and psychological wellbeing: the effects of political conflict exposure. Polit. Psychol. 36, 75–92. doi: 10.1111/pops.12073

[ref69] Smail J. R. (1965). Agricultural involution: the process of ecological change in Indonesia. By Clifford Geertz. University of California Press, Berkeley and Los Angeles, 1963. Pp. 176. Bibliography, Index. J. Southeast Asian History 6, 158–161. doi: 10.1017/S021778110000209X

[ref70] Smola K. W. Sutton C. D. (2002). Generational differences: revisiting generational work values for the new millennium. J. Org. Behav. 23, 363–382. doi: 10.1002/job.147

[ref71] Stott C. Drury J. Reicher S. (2017). On the role of a social identity analysis in articulating structure and collective action: the 2011 riots in Tottenham and Hackney. Br. J. Criminol. 57, 964–981. doi: 10.1093/bjc/azw036

[ref72] Stout N. (2016). # indebted: disciplining the moral valence of mortgage debt online. Cult. Anthropol. 31, 82–106. doi: 10.14506/ca31.1.05

[ref73] Striphas T. (2015). Algorithmic culture. Eur. J. Cult. Stud. 18, 395–412. doi: 10.1177/1367549415577392, PMID: 37125808

[ref74] Sun J. Cheng D. (2018). China’s generation gap. New York: Routledge.

[ref75] Sunstein C. R. (2018). #Republic: divided democracy in the age of social media. Princeton, NJ: Princeton University Press.

[ref76] Tajfel H. Turner J. C. Austin W. G. Worchel S. (1979). An integrative theory of intergroup conflict. Organ. Identity 56:9780203505984-16.

[ref77] Turner J. C. Brown R. J. Tajfel H. (1979). Social comparison and group interest in ingroup favouritism. Eur. J. Soc. Psychol. 9, 187–204. doi: 10.1002/ejsp.2420090207

[ref79] Turner J. C. Reynolds K. J. (1987). Rediscovering the social group: a self-categorization theory. Oxford & New York: Blackwell

[ref80] Wang Qn Ge Sf. (2020). Interview with Xiang Biao: involution, an endless competition. PengPai. Available at: https://www.thepaper.cn/newsDetail_forward_9648585.

[ref81] Wood S. (2005). Spanning the generation gap in the workplace. Am. Water Works Assoc. 97, 86–87. doi: 10.1002/j.1551-8833.2005.tb10888.x, PMID: 32469240

[ref82] Yang Y. Schulhofer-Wohl S. Fu W. J. Land K. C. (2008). The intrinsic estimator for age-period-cohort analysis: what it is and how to use it. Am. J. Sociol. 113, 1697–1736. doi: 10.1086/587154, PMID: 36849004

[ref83] Yu R. Cheung O. Leung J. Tong C. Lau K. Cheung J.. (2019). Is neighbourhood social cohesion associated with subjective well-being for older Chinese people? The neighbourhood social cohesion study. BMJ Open 9:e023332. doi: 10.1136/bmjopen-2018-023332, PMID: 31079078 PMC6530414

[ref84] Zemke R. Raines C. Filipczak B. (2000). Generations at work: managing the clash of veterans, boomers, Xers, and Nexters in your workplace. Train. Dev. 54:60.

[ref85] Zhu J. Weng F. Zhuang M. Lu X. Tan X. Lin S.. (2022). Revealing public opinion towards the COVID-19 vaccine with Weibo data in China: BertFDA-based model. Int. J. Environ. Res. Public Health 19:13248. doi: 10.3390/ijerph192013248, PMID: 36293828 PMC9602858

